# Treatment of Old Fractures by Division of Tendons

**Published:** 1842-08

**Authors:** 


*Treatment of Old Fractures by Division of Tendons.*—Prof. Diffenbach has several times, in old cases of fracture of the patella or the olecranon, where the portions were dragged far apart, divided the adjacent tendons so as to be able to bring the portions together, and, by friction of them one upon the other, to excite such action as might end in the formation of a shorter and firmer bond of union. In some cases, considerable benefit was obtained after all other means had failed; in others, the result was negative. Two examples are detailed; in one, an old ununited fracture of the ulna, he divided the tendon of the triceps, fixed the upper portion of the bone in its right place by a bandage, and every fourteen days rubbed it well against the lower one: in three months the union was firm. In another example, an old, distantly united fracture of the patella, he divided the ligamentum patellæ and the rectus femoris about three inches above the patella; then, by an appropriate bandage, and constantly drawing the separated portions more closely together, he obtained, at the end of some months, a complete hardening of the interposed substance, and a considerable amelioration of the patient's state.—*British and For. Med. Rev.*, from *Casper's Wochenschrift*, Oct. 2, 1841.

